# Red Blood Cell Membrane Fatty Acid Composition, Dietary Fatty Acid Intake and Diet Quality as Predictors of Inflammation in a Group of Australian Adults

**DOI:** 10.3390/nu15102405

**Published:** 2023-05-21

**Authors:** Erin D. Clarke, Jordan Stanford, Jessica J. A. Ferguson, Lisa G. Wood, Clare E. Collins

**Affiliations:** 1 School of Health Sciences, College of Health Medicine and Wellbeing, The University of Newcastle, Callaghan, NSW 2308, Australia; 2 Hunter Medical Research Institute (HMRI) Food and Nutrition Research Program, HMRI, New Lambton Heights, NSW 2305, Australia; 3 Hunter Medical Research Institute, New Lambton Heights, NSW 2305, Australia; 4 School of Biomedical Sciences and Pharmacy, College of Health, Medicine and Wellbeing, University of Newcastle, Callaghan, NSW 2308, Australia

**Keywords:** fatty acids, diet quality, inflammation

## Abstract

Evidence suggests that diet can play a role in modulating systemic inflammation. This study aims to examine the relationship between fatty acids (FAs) (self-reported dietary intake and red blood cell (RBC) membrane fatty acid concentrations), three diet quality scores, and the plasma concentrations of inflammatory markers (interleukin-6, IL-6; tumour necrosis factor alpha, TNF-α; and C-reactive protein, CRP) in a group of Australian adults (*n* = 92). Data were collected on their demographic characteristics, health status, supplement intake, dietary intake, RBC-FAs and plasma inflammatory markers over a nine-month period. Mixed-effects models were used to determine the relationship between RBC-FAs, dietary intake of FAs, diet quality scores and inflammatory markers to determine which variable most strongly predicted systemic inflammation. A significant association was identified between dietary saturated fat intake and TNF-α (β = 0.01, *p* < 0.05). An association was also identified between RBC membrane saturated fatty acids (SFA) and CRP (β = 0.55, *p* < 0.05). Inverse associations were identified between RBC membrane monounsaturated fatty acids (MUFAs) (β = −0.88, *p* < 0.01), dietary polyunsaturated fatty acids (PUFAs) (β = −0.21, *p* < 0.05) and CRP, and the Australian Eating Survey Modified Mediterranean Diet (AES-MED) score and IL-6 (β = −0.21, *p* < 0.05). In summary, using both objective and subjective measures of fat intake and diet quality, our study has confirmed a positive association between saturated fat and inflammation, while inverse associations were observed between MUFAs, PUFAs, the Mediterranean diet, and inflammation. Our results provide further evidence that manipulating diet quality, in particular fatty acid intake, may be useful for reducing chronic systemic inflammation.

## 1. Introduction

Inflammation is a vital process that supports recovery from injury and maintains overall health [1]. However, persistent low-grade inflammation without acute triggers contributes to a range of chronic diseases, such as hypertension, cardiovascular disease (CVD), type 2 diabetes (T2D) and cancer [2]. Measuring serum inflammatory biomarkers, such as C-reactive protein (CRP), interleukin 6 (IL-6) and tumour necrosis factor α (TNF-α), can provide insights into an individual’s inflammatory status [3].

Diet can play a role in modulating inflammatory status. Previous studies have primarily examined the impact of individual vitamins and nutrients on systemic inflammation [4]. Dietary fats have been suggested to have a direct effect on inflammation [5]. Saturated fatty acids (SFAs) are a necessary component of bacterial endotoxins such as lipopolysaccharide (LPS) [6]. Macrophages and other innate immune cells contain receptors (toll-like receptor 4, TLR4) that recognise LPS, and the subsequent binding of saturated fatty acids to TLR4 results in the activation of a transcription factor (NF-κB) that initiates the expression of diverse pro-inflammatory cytokines, including TNF-α, IL-1, IL-6 and IL-8 [6,7].

The analysis of individual dietary components in isolation fails to consider the intricate nature of dietary patterns and the collective and synergistic impact of various dietary factors, which may carry more significant effects. To assess dietary patterns, a priori diet scores, such as the Dietary Inflammatory Index (DII), or established patterns of food consumption, such as the Mediterranean diet score, are used to measure adherence and associations with positive health outcomes [8]. Healthy dietary patterns, such as the Mediterranean diet, which is low in saturated and trans-fats but abundant in monounsaturated fatty acids (MUFAs), polyunsaturated fatty acids (PUFAs), vitamins C and E, and plant-derived phenolic compounds, have been shown to improve circulating inflammatory biomarkers and result in better glycaemic control, reduced cardiovascular morbidity and reduced mortality [9].

This study aims to examine the relationship between fatty acid intake (assessed by self-reported dietary intake and red blood cell (RBC) membrane fatty acid concentrations), diet quality (evaluated using the Mediterranean diet score, Australian Recommended Food Score and Dietary Inflammatory Index) and plasma concentrations of inflammatory markers (IL-6, TNF-α and CRP) in a group of Australian adults. We hypothesised that higher dietary intakes of MUFAs and PUFAs, higher diet quality scores and a more anti-inflammatory dietary pattern are associated with lower levels of systemic inflammation as measured by the aforementioned inflammatory markers. It is also anticipated that concentrations of MUFAs and PUFAs in red blood cell membranes may also be associated with lower levels of systemic inflammatory markers. Conversely, higher levels of saturated fatty acids, measured through self-reported dietary intake and an RBC analysis, are associated with higher levels of systemic inflammation as measured by these same markers.

## 2. Materials and Methods

### 2.1. Participants and Ethics

Participants were recruited from the Newcastle region, NSW Australia between September 2019 and March 2020 via media releases, flyers and other local media. Eligible participants were ≥18 years old, had a stable weight (±4 kg) for the last 2 months, had access to the internet and were able to travel to the University of Newcastle for data collection sessions. Participants were excluded if they were pregnant, breastfeeding, were trying to conceive, had an electronic device such as a pacemaker or cochlear implant, were taking medications or supplements known to affect metabolic rate/weight/fluid balance, had food allergies or intolerances, claustrophobia, or a chronic medical condition, such as heart failure, kidney disease, liver disease or similar condition.

Ethics was approved by the University of Newcastle Human Research Ethics Committee (H-2019-0147) and the trial was registered with Australian New Zealand Clinical Trials Registry (ANZCTR-12619001415190).

### 2.2. Measurement Sessions

Measurement sessions were conducted up to 9 months apart and included the collection of data on anthropometric measures, demographic characteristics, dietary intake and blood collection for the analysis of RBC fatty acids (RBC-FAs) and inflammatory markers.

Anthropometric measures included height and weight (Inbody 270, Seoul, Republic of Korea) which were used to calculate body mass index (BMI). Body composition was measured using bioelectrical impedance analysis (Inbody 270, Seoul, Republic of Korea).

Questionnaires were completed using Qualtrics XM System (Provo, UT, USA). Demographic data, including age, smoking status, medications, education level, employment and health status, were all self-reported.

### 2.3. Dietary Intake and Diet Quality Indexes

Dietary intake was assessed using the Australian Eating Survey (AES) food frequency questionnaire completed using Qualtrics XM System (Provo, UT, USA). The AES contains 135 questions of which 120 items are related to dietary intake over the past 3 months and has been validated previously for use in adults [10] and against RBC-FAs [11]. The AES uses the AUSNUT 2011-13 food and nutrient composition data to calculate nutrient intakes, such as fatty acids [12]. Fatty acid intakes reported include total saturated, monounsaturated, polyunsaturated, and omega-3 polyunsaturated fat intakes calculated from the AES food frequency questionnaire.

#### 2.3.1. The Australian Recommended Food Score (ARFS) Diet Quality Index

The Australian Recommended Food Score (ARFS) is a diet quality index calculated from a sub-set of 70 questions from the AES food frequency questionnaire [13]. The diet quality score is calculated using the ARFS ranging from 0 to 73 points. Higher scores relate to a greater intake of nutrient-rich core food intakes that align with the Australian Dietary Guidelines, such as fruit, vegetables, meat, plant-based proteins, breads and cereals, dairy foods, water and spreads/sauces.

#### 2.3.2. AES Modified Mediterranean Diet Score (AES-MED)

A Mediterranean diet score was created from AES responses based on the scoring by Sofi et al. [14] minus two points for olive oil as this is not collected in the AES. Therefore, the scoring for the AES-MED ranges from 0 to 16. Higher scores suggest higher diet quality and greater alignment with a Mediterranean dietary pattern, including greater intakes of fruits, vegetables, legumes, fish and cereals, and lower intakes of meat and meat products, dairy and moderate alcohol intake. Food groupings were kept the same as those defined in the AES, except fish was separated from the meat group due to being separate for the scoring of the Mediterranean diet.

#### 2.3.3. AES Dietary Inflammatory Index (AES-DII)

A Dietary Inflammatory Index (AES-DII) score was created based on Shivappa et al.’s [15] methodology and applied to the AES. A total of 27 nutrients were used to create the AES-DII score; this included alcohol, beta-carotene, caffeine, carbohydrates, cholesterol, energy, total fat, fibre, folic acid, iron, magnesium, monounsaturated fatty acids, niacin, omega 3, omega 6, protein, polyunsaturated fatty acids, riboflavin, saturated fat, selenium, thiamin, trans fats, vitamin B12, vitamin B6, vitamin C, vitamin E and zinc. Firstly, each nutrient unit of the report was checked to ensure it matched that used on the DII and was adjusted by multiplying or dividing as necessary. Then the five steps outlined by Shivappa et al. were followed to create the AES-DII score using available nutrients. These steps included the following: 1. Subtracting the standard mean from the amount reported; 2. Dividing this value by the standard deviation; 3. Adjusting for right skewedness by changing z-score to percentile, doubling it and subtracting one; 4. Multiplying by the overall inflammatory effect score; and 5. Summing all scores to create an overall DII score. Positive AES-DII scores (maximum score of +7.98) are pro-inflammatory, and negative scores (maximum of −8.87) are anti-inflammatory.

### 2.4. Red Blood Cell Fatty Acid Analysis

Fasted, venous blood samples were collected by a trained phlebotomist at an accredited pathology service (NSW Health Pathology—North located at the University of Newcastle). Samples were collected at data collection sessions in EDTA tubes before being separated into plasma and RBC fractions and stored at −80 °C prior to analysis.

The RBC fractions were thawed and lysed. The membranes were solubilised and purified by adding 12 mL of hypotonic tris buffer and 12 mL of 0.25 M glucose solution to 500 µL of RBCs, then centrifuged at 10,000 rpm at 4 °C for 10 min. After centrifugation, the supernatant was collected and discarded before the process above was repeated twice more, with the centrifuge set at 12,000 rpm at 4 °C for 10 min and then at 15,000 rpm at 4 °C for 20 min. A total of 250 µL of glucose solution and 250 µL of tris buffer solution were added to resuspend the pellet before being stored at −80 °C prior to analysis.

The RBC membrane fatty acid concentrations were determined using a previously established method [16,17]. Briefly, a methanol/toluene (4:1 *v*/*v*) mixture containing C13:0 and C19:0 as internal standards and BHT (0.12 g/L) was added to the membrane suspension. Acetyl chloride was added dropwise while vortexing, and samples were then heated at 100 °C for one hour to methylate the fatty acids. To stop the reaction, the sample was cooled, then 6% K2CO3 was added. To separate the layers, the sample was centrifuged at 3000 rpm at 4 °C for 10 min. The upper toluene layer was used for gas chromatography (GC) analysis of the fatty acid methyl esters. Analysis was undertaken using a 30 m × 0.25 mm (DB-225) fused carbon silica column coated with cyanopropylphenyl (J & W Scientific, Folsom, CA, USA). Fatty acid methyl ester peaks were identified by comparing their retention times with those of standard mixture of fatty acid methyl esters (GLC-462, Nu-Chek Prep Inc., Elysian, MN, USA) and quantified using a Hewlett Packard 6890 Series gas chromatograph with a flame ionization detector and Chemstations software (version A.04.02, Hewlett Packard, Palo Alto, CA, USA).

### 2.5. Inflammatory Marker Analysis

Plasma IL-6, TNF-α (R&D Systems, Minneapolis, MN, USA) and CRP (MP-Biomedicals, Seven Hills, NSW, Australia) plasma were analysed by high-sensitivity enzyme-linked immunosorbent assay (ELISA) kits as per manufacturer’s specifications as described previously [18].

### 2.6. Statistical Analysis

Statistical analyses were conducted using Stata version 14.2 (StataCorp, College Station, TX, USA). Firstly, data were checked for normality. Normally distributed data were presented as mean (SD), qualitative variables were summarised by n (%) and skewed data was presented as median (IQR). Participants were excluded if they had missing data, and one person was excluded from the analysis for taking a medication that inhibits TNF-α. Mixed-effects models were run between RBC-FAs, dietary fatty acids, diet quality scores and inflammatory markers to determine which dietary factor explained more of the relationship with inflammation. Mixed-effects models were employed in the study due to the use of repeated measures data, enabling the analysis of observations taken from the same individuals over time, while accounting for within-subject autocorrelation. Mixed-effects models were reported as unadjusted and adjusted by time and for known confounders (age, BMI, sex, anti-inflammatory supplement intake, presence of inflammatory condition and smoking status). Bonferroni correction was applied to account for the multiple comparisons.

## 3. Results

**Figure 1:**
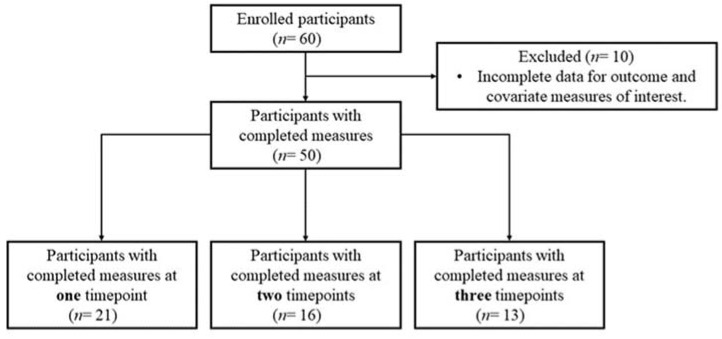
Participant flow chart.

The cohort included in the current study was made up of 50 individuals, for which a total of 92 measures were taken. The repeated measures data were available for 29 participants over nine months (Figure 1). The participants were mostly female, and only three participants reported a pro-inflammatory condition. The participant characteristics are summarised in Table 1.

The dietary fatty acid intake, diet quality scores, RBC-FAs and inflammatory markers are reported in Table 2. Overall, the diet quality scores were moderate, with an average ARFS score of 40.5 out of a maximum of 73 points. The median AES-MED score was 9.0 out of a maximum of 16, and the median AES-DII score was anti-inflammatory at −0.98.

### 3.1. The Relationship between Inflammation and Fatty Acids Measures through Diet and Red Blood Cell Membranes

**Figure 2:**
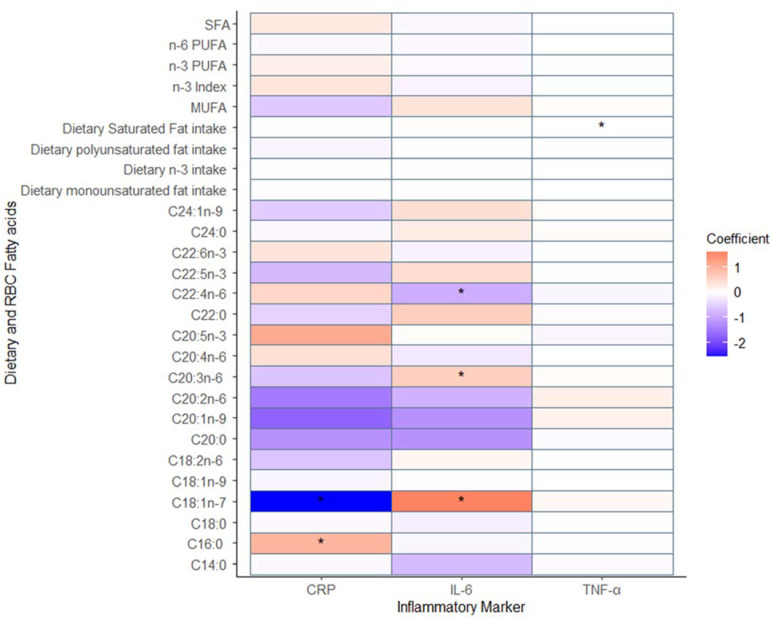
Heat map of the multiple-adjustment mixed-effect models showing the relationship between red blood cells, dietary fatty acids and inflammatory markers. * denotes relationship is statistically significant.

No statistically significant relationships were identified between RBC membrane or dietary fatty acids and IL-6, as shown in Figure 2 and Supplementary Table S1. A statistically significant inflammatory relationship was identified between dietary saturated fat intake and TNF-α (β = 0.01, *p* < 0.05), which remained significant in the adjusted models. A significant inflammatory relationship was also identified between RBC membrane SFA and CRP (β = 0.55, *p* < 0.05); this only remained significant when adjusting for time. An anti-inflammatory relationship was identified between RBC membrane MUFAs and CRP; this remained significant when adjusting for time but not all confounders (β = −0.88, *p* < 0.01). Lastly, self-reported dietary PUFA intake was identified to have a significant anti-inflammatory relationship with CRP (β = −0.21, *p* < 0.05); again, this did not remain significant in the fully adjusted model.

The relationship between individual RBC fatty acids and inflammation was also examined (Supplementary Table S2). Significant relationships were identified between IL-6 and C18:1n-7 (vaccenic acid, β = 1.61, *p* < 0.05), C20:3n-6 (homo-γ-linolenic, β = 0.63, *p* < 0.05), and C22:4n-6 (adrenic acid, β = −0.89, *p* < 0.05). No significant relationships were identified with TNF-α and individual RBC fatty acids. Significant relationships were also identified between CRP and C16:0 (palmitic acid, β = 0.98, *p* < 0.05), C18:1n-7 (vaccenic acid, β = −2.56, *p* < 0.05), C18:2n-6 (linoleic acid, β = −0.79, *p* = 0.01), C20:3n-6 (homo-γ-linolenic, β = −0.94, *p* < 0.05), C20:4n-6 (arachidonic acid, β = 0.59, *p* < 0.02), and C24:1n-9 (nervonic acid, β = −1.01, *p* < 0.05).

### 3.2. The Relationship between Inflammation and Dietary Patterns

In the unadjusted model, a statistically significant anti-inflammatory relationship was identified between IL-6 and the AES-MED score (β = −0.21, *p* < 0.05); these results remained significant when adjusted for time only, as shown in Table 3. These results suggest that for each one-point increase in AES-MED score, there is a 21 pg/mL reduction in IL-6.

## 4. Discussion

The current study aimed to determine the relationship between fatty acids, diet quality and inflammatory markers (IL-6, TNF-α and CRP) in a sample of Australian adults. We observed significant pro-inflammatory relationships between saturated fatty acids and inflammation, as well as anti-inflammatory relationships between RBC-n-3 PUFAs, RBC-MUFAs, RBC-n-3 Index and Mediterranean diet scores.

Saturated fat has been implicated in promoting inflammatory pathways through processes such as the activation of TLR4 and, as a result, can contribute to increases in systematic inflammation [19]. The current study identified a significant relationship between dietary SFA and the inflammatory marker TNF-α. Marginal associations between RBC-SFAs, CRP and IL-6, but not TNF-α, were observed in a similar population of generally healthy participants [20]. Overall, systematic reviews report inconsistent results regarding the relationship between SFA and inflammation, including CRP, TNF-α and IL-6 [20,21]. Only one study reported a weak but significant relationship between plasma SFA and IL-6 (β = 0.02, *p* = 0.01) [22]. While the current study identified a significant relationship between dietary SFA and TNF-α, each 10 g increase in SFA intake resulted in a 0.01 pg/mL increase in TNF-α, which is not clinically significant. Researchers have emphasised that while the relationship between SFAs and inflammation has been well documented in human cell lines and animal studies, further research is required to fully understand the association in human populations [23].

Evidence suggests that replacing saturated fats with MUFAs or PUFAs can reduce inflammation, reduce endoplasmic reticulum stress and stimulate the expression of the adiponectin gene, which in turn can reduce the production of IL-6 and TNF-α [19,23]. Findings from the current study identified a significant anti-inflammatory relationship between dietary PUFAs and CRP. Previous research in large observational studies has reported similar findings: higher dietary intakes of PUFAs were associated with lower CRP levels [24,25]. Although some studies suggest this may be driven mainly by higher intakes of n-6 PUFAs, the data are heterogeneous [24,26]. Supplementation with n-3 PUFAs has been suggested to reduce systemic inflammation; however, findings are conflicting [27,28]. The anti-inflammatory effects demonstrated in healthy individuals have been reported following LC n-3 PUFA supplementation [28], and since the AES does not differentiate between various types of n-3 PUFAs in the diet, this may explain why an association between n-3 PUFAs and inflammatory markers was not demonstrated in the current study. While the association between dietary PUFAs and CRP did not remain significant in the final adjusted model, these findings are supported by prior research and indicate that dietary intervention studies are warranted to examine this relationship further. Similarly, a significant inverse relationship was observed between total RBC-MUFAs and CRP levels in the current study. These findings are similar to previous cross-sectional studies, which have reported an anti-inflammatory relationship between MUFAs and CRP [22,29]. This relationship may have been driven by the individual MUFAs C18:1n-7 (vaccenic acid) and C24:1n-9 (nervonic acid), which were inversely associated with CRP levels in the adjusted models. The positive association between CRP levels and both C16:0 (palmitic acid) and C20:4n-6 (arachidonic acid) as well as the inverse association between CRP levels and C18:2n-6 (linoleic acid) are similar to those reported in a large observational study of healthy Canadian adults [30]. Although the latter study did not investigate IL-6 or TNF-α concentrations, they also reported a positive inflammatory relationship with C20:3n-6 (homo-γ-linolenic) and CRP, which was found in the current study, though with IL-6 levels. Future studies are warranted to confirm these findings and assess the relationship between RBC fatty acids beyond their total fatty acid class to delineate which individual fatty acids (and thus specific dietary sources) might be driving this relationship. Although some diet-derived fatty acids were significantly associated with inflammatory markers in the current study (SFA and TNF-α; PUFA and CRP), this was not replicated in the fatty acid results derived from RBCs. The degree of endogenous synthesis may account for these discrepancies [31,32], whereby endogenous fatty acid synthesis may contribute in part to the proportion of fatty acids in RBCs. The influence of potential confounders, such as age, BMI, sex, time, anti-inflammatory supplement intake, inflammatory conditions and smoking status, appeared to influence the relationship between RBC-SFA and RBC-MUFA, as well as PUFA and CRP in this sample, indicating that future studies should consider performing sub-group analyses and/or specifically target certain population groups when aiming to explore the association between fatty acids and CRP.

Healthy dietary patterns, including those characterised by higher intakes of unsaturated fats, have been found to be inversely associated with inflammation [8,33]. Of the three dietary indexes explored, only the AES-MED score had a significant anti-inflammatory relationship. While other dietary patterns, such as the DII, have been found to be anti-inflammatory at more negative scores, a recent systematic review of observational studies identified a third of analyses using the DII found no association with inflammatory markers, suggesting that this result is not always consistent [33]. The majority of systematic reviews in observational and intervention studies have shown the Mediterranean diet to have anti-inflammatory properties, indicated by inverse associations between the MED score and inflammatory biomarkers [9,33,34]. These reviews identified that a Mediterranean diet was mostly inversely associated with CRP; however, like the current study, lower IL-6 concentrations were also found to be associated with higher Mediterranean diet scores [9,33,34]. A Mediterranean diet is high in foods rich in MUFAs and low in foods rich in SFAs; these defining characteristics of the Mediterranean diet as well as the high intakes of fruits, vegetables and whole grains likely contribute to the anti-inflammatory effects identified with this dietary pattern. Additionally, a Mediterranean diet promotes a high consumption of olive oil, which is predominantly MUFA and contains several phenolic compounds, including oleuropein, hydroxytyrosol and tyrosol, which have been found to have antioxidant and anti-inflammatory properties [35,36].

There are several strengths and limitations to this study. The strengths of the current study include the combined use of objective (RBC membrane) and self-reported (AES) methods of measuring fatty acids. This paper also explored the relationship with three dietary patterns, one which is designed specifically for the Australian population based on dietary guidelines (ARFS) and two patterns (AES-MED and AES-DII) which have strong evidence for being used to identify anti-inflammatory dietary patterns [33]. Additionally, this study used a combination of single nutrients and dietary patterns to provide a real-world scenario in which foods are consumed as more than single nutrients [23]. The limitations of the current study include that it is an observational study and therefore cannot demonstrate causality, nor account for the de novo synthesis of FAs. It was also outside the scope of this study to explore different subclasses of fatty acids derived from the diet. Whilst the current study presents confirmatory findings of previous results, future research is warranted to examine this relationship in larger samples, and across various populations. Furthermore, future studies exploring the relationship between dietary fatty acids, their subclasses and subclinical inflammation need to consider how an individual’s endogenous fatty acid synthesis capacity might modify this relationship [37]. Lastly, the population in the current study had a higher BMI which was found to be associated with inflammation (not reported); this may explain why the results no longer remained significant when BMI was included in the model. Furthermore, due to the intricate interplay between diet and BMI, in this particular cohort, disentangling their respective effects is challenging.

## 5. Conclusions

In summary, using both objective and subjective measures of fat intake and diet quality, our study has confirmed a positive association between saturated fat and inflammation, while inverse associations were observed between MUFAs, PUFAs, Mediterranean diet and inflammation. Our results provide further evidence that manipulating diet quality, in particular fatty acid intake, may be useful for reducing chronic systemic inflammation.

## Figures and Tables

**Table 1:** Participant baseline demographic characteristics (*n* = 92).

Characteristic	N (%)	Mean (SD)
**Gender**		
Female	63 (68)	
Male	29 (32)	
**Age (years)**		37.4 (16.6)
**BMI (kg/m^2^)**		25.2 (5.2)
**Smoking status**		
Not at all	85 (92)	
Yes, less than weekly	5 (5)	
Yes, at least weekly	1 (1)	
Yes, daily	1 (1)	
**Intake of anti-inflammatory supplements ^(a)^**		
Yes	7 (8)	
No	85 (92)	
**Presence of inflammatory condition ^(b)^**		
Yes	3 (3)	
No	89 (97)	

^(a)^ Anti-inflammatory supplements included long-chain omega-3 PUFAs, curcumin and coenzyme Q10. ^(b)^ Inflammatory conditions refer to arthritis only in this cohort.

**Table 2:** Dietary intakes, red blood cell fatty acid concentrations and inflammatory markers (*n* = 92).

	Mean (SD)	Median [IQR]
**AES Fatty acid intakes (g/day)**		
Saturated fat intake		27.52 [14.95]
Monounsaturated fat intake		32.24 [15.76]
Polyunsaturated fat intake		11.10 [5.31]
**Diet Quality Scores**	40.52 (13.02)	
ARFS (0–73)		
AES-MED (0–16)		9.00 [3.00]
AES-DII		−0.98 [1.55]
**Red blood cell fatty acids (%)**		
C14:0	0.12 (0.36)	
C16:0		21.45 [0.95]
C18:0		16.43 [1.19]
C18:1n-9	13.25 (1.19)	
C18:1n-7		1.02 [0.23]
C18:2n-6		9.98 [1.76]
C20:0	0.06 (1.89)	
C20:1n-9	0.02 (0.10)	
C20:2n-6	0.02 (0.11)	
C20:3n-6		1.55 [0.93]
C20:4n-6	15.48 (1.68)	
C20:5n-3		0.95 [0.41]
C22:0		1.47 [0.27]
C22:4n-6	3.20 (0.67)	
C22:5n-3	2.35 (0.54)	
C22:6n-3	5.19 (1.17)	
C24:0		3.42 [1.00]
C24:1n-9		3.89 [1.15]
Total Saturated fat		43.02 [1.73]
Total Monounsaturated fat		17.89 [1.84]
Total n-6 Polyunsaturated fat	30.22 (2.09)	
Total n-3 Polyunsaturated fat		8.49 [1.93]
n-3 Index	6.14 (1.50)	
**Inflammatory markers**		
IL-6 (pg/mL)		1.17 [0.82]
TNF-α (pg/mL)		0.92 [0.46]
CRP (mg/L)		1.51 [2.36]

Data are presented for each participant when available, totalling 92 datapoints. AES, Australian Eating Survey; AES-DII, AES Dietary Inflammatory Index; AES-MED, Australian Eating Survey Modified Mediterranean Diet Score; ARFS, Australian Recommended Food Score; CRP, C-reactive protein; IL-6, interleukin 6; and TNF-α, tumour necrosis factor α. Individual fatty acids below detectable limits: C10:0, C12:0, C12:1, C14:1n-7, C16:1n-7, C18:3n-6 (Gamma), C18:3n-3 (Alpha), C20:3n-3 (DH-14-17), C22:1n-9 and C22:2n-6.

**Table 3:** Adjusted and unadjusted mixed-effects models showing the relationship between diet quality scores and inflammatory markers.

	Unadjusted		Simple Adjustment ^(a)^		Multiple Adjustments ^(b)^	
	β (95% CI)	*p*-Value	β (95% CI)	*p*-Value	β (95% CI)	*p*-Value
**IL-6 and Diet Quality**						
ARFS	0.00 (−0.04, 0.04)	0.89	0.00 (−0.05, 0.06)	0.91	0.02 (−0.04, 0.09)	0.47
AES-MED	**−0.21** (**−0.43, −0.002**)	**0.048**	**−0.25** (**−0.46, −0.04**)	**0.02**	−0.23 (−0.49, 0.03)	0.08
AES-DII	0.00 (−0.42, 0.42)	0.998	0.03 (−0.39, 0.45)	0.88	−0.14 (−0.66, 0.39)	0.61
**TNF-α and Diet Quality**						
ARFS	0.00 (−0.004, 0.004)	0.98	0.00 (−0.01, 0.01)	0.77	0.01 (−0.001, 0.02)	0.09
AES-MED	0.00 (−0.03, 0.03)	0.87	−0.01 (−0.04, 0.02)	0.67	−0.02 (−0.06, 0.01)	0.19
AES-DII	−0.02 (−0.08, 0.04)	0.47	−0.02 (−0.08, 0.04)	0.49	−0.04 (−0.11, 0.03)	0.31
**CRP and Diet Quality**						
ARFS	−0.05 (−0.10, 0.01)	0.11	−0.07 (−0.15, 0.02)	0.11	−0.04 (−0.14, 0.07)	0.48
AES-MED	−0.10 (−0.43, 0.22)	0.52	−0.13 (−0.45, 0.19)	0.43	−0.03 (−0.47, 0.40)	0.89
AES-DII	0.38 (−0.23, 1.00)	0.22	0.41 (−0.21, 1.02)	0.20	0.12 (−0.72, 0.95)	0.78

Data are presented for mixed-effects models with beta-coefficients, 95% CI and *p*-values. Findings with *p* < 0.05 are considered statistically significant and bolded. ^(a)^ adjusted for time only. ^(b)^ adjusted for age, BMI, sex, time, anti-inflammatory supplement intake, inflammatory conditions and smoking status. AES, Australian Eating Survey; AES-DII, AES Dietary Inflammatory Index; AES-MED, Australian Eating Survey Modified Mediterranean Diet Score; ARFS, Australian Recommended Food Score; CRP, C-reactive protein; IL-6, interleukin 6; and TNF-α, tumour necrosis factor α.

## Data Availability

The data presented in this study are available on request from the corresponding author. The data are not publicly available as ethics approval was not granted for this.

## Supplementary Materials

The following supporting information can be downloaded at: https://www.mdpi.com/article/10.3390/nu15102405/s1, Table S1: Adjusted and unadjusted mixed-effects models showing the relationship between red blood cell and dietary fatty acids and inflammatory markers; Table S2: Adjusted and unadjusted mixed-effects models showing the relationship between individual red blood cell membrane fatty acids and inflammatory markers.

## References

[1] Ridker P.M. (2016). Residual inflammatory risk: Addressing the obverse side of the atherosclerosis prevention coin. Eur. Heart J..

[2] Hotamisligil G.S. (2006). Inflammation and metabolic disorders. Nature.

[3] Kaptoge S., Di Angelantonio E., Lowe G., Pepys M.B., Thompson S.G., Collins R., Danesh J., Emerging Risk Factors Collaboration (2010). C-reactive protein concentration and risk of coronary heart disease, stroke, and mortality: An individual participant meta-analysis. Lancet.

[4] Calder P.C., Yaqoob P. (2013). Diet, Immunity and Inflammation.

[5] Calder P.C., Ahluwalia N., Brouns F., Buetler T., Clement K., Cunningham K., Esposito K., Jönsson L.S., Kolb H., Lansink M. (2011). Dietary factors and low-grade inflammation in relation to overweight and obesity. Br. J. Nutr..

[6] Calder P.C. (2012). Mechanisms of Action of (n-3) Fatty Acids. J. Nutr..

[7] Fritsche K. (2006). Fatty Acids as Modulators of the Immune Response. Annu. Rev. Nutr..

[8] Aleksandrova K., Koelman L., Rodrigues C.E. (2021). Dietary patterns and biomarkers of oxidative stress and inflammation: A systematic review of observational and intervention studies. Redox Biol..

[9] Schwingshackl L., Hoffmann G. (2014). Mediterranean dietary pattern, inflammation and endothelial function: A systematic review and meta-analysis of intervention trials. Nutr. Metab. Cardiovasc. Dis..

[10] Collins C.E., Boggess M.M., Watson J.F., Guest M., Duncanson K., Pezdirc K., Rollo M., Hutchesson M.J., Burrows T.L. (2014). Reproducibility and comparative validity of a food frequency questionnaire for Australian adults. Clin. Nutr..

[11] Schumacher T.L., Burrows T.L., Rollo M.E., Wood L.G., Callister R., Collins C.E. (2016). Comparison of fatty acid intakes assessed by a cardiovascular-specific food frequency questionnaire with red blood cell membrane fatty acids in hyperlipidaemic Australian adults: A validation study. Eur. J. Clin. Nutr..

[12] Food Standards Australia and New Zealand (2019). AUSNUT 2011-13 Food Nutrient Database. https://www.foodstandards.gov.au/science/monitoringnutrients/ausnut/ausnutdatafiles/pages/foodnutrient.aspx.

[13] Collins C.E., Burrows T.L., Rollo M.E., Boggess M.M., Watson J.F., Guest M., Duncanson K., Pezdirc K., Hutchesson M.J. (2015). The Comparative Validity and Reproducibility of a Diet Quality Index for Adults: The Australian Recommended Food Score. Nutrients.

[14] Sofi F., Macchi C., Abbate R., Gensini G.F., Casini A. (2013). Mediterranean diet and health status: An updated meta-analysis and a proposal for a literature-based adherence score. Public Health Nutr..

[15] Shivappa N., Steck S.E., Hurley T.G., Hussey J.R., Hébert J.R. (2014). Designing and developing a literature-derived, population-based dietary inflammatory index. Public Health Nutr..

[16] Stoodley I., Garg M., Scott H., Macdonald-Wicks L., Berthon B., Wood L. (2019). Higher Omega-3 Index Is Associated with Better Asthma Control and Lower Medication Dose: A Cross-Sectional Study. Nutrients.

[17] Lepage G., Roy C.C. (1986). Direct transesterification of all classes of lipids in a one-step reaction. J. Lipid Res..

[18] Berthon B.S., McLoughlin R.F., Jensen M.E., Hosseini B., Williams E.J., Baines K.J., Taylor S.L., Rogers G.B., Ivey K.L., Morten M. (2021). The effects of increasing fruit and vegetable intake in children with asthma: A randomized controlled trial. Clin. Exp. Allergy.

[19] Fritsche K.L. (2015). The Science of Fatty Acids and Inflammation. Adv. Nutr. Int. Rev. J..

[20] Mu L., Mukamal K.J., Naqvi A.Z. (2014). Erythrocyte saturated fatty acids and systemic inflammation in adults. Nutrition.

[21] Santos S., Oliveira A., Lopes C. (2013). Systematic review of saturated fatty acids on inflammation and circulating levels of adipokines. Nutr. Res..

[22] Kalogeropoulos N., Panagiotakos D.B., Pitsavos C., Chrysohoou C., Rousinou G., Toutouza M., Stefanidis C. (2010). Unsaturated fatty acids are inversely associated and n-6/n-3 ratios are positively related to inflammation and coagulation markers in plasma of apparently healthy adults. Clin. Chim. Acta..

[23] Grosso G., Laudisio D., Frias-Toral E., Barrea L., Muscogiuri G., Savastano S., Colao A. (2022). Anti-Inflammatory Nutrients and Obesity-Associated Metabolic-Inflammation: State of the Art and Future Direction. Nutrients.

[24] Muka T., Jong J.C.K.-D., Hofman A., Dehghan A., Rivadeneira F., Franco O.H. (2015). Polyunsaturated Fatty Acids and Serum C-Reactive Protein: The Rotterdam Study. Am. J. Epidemiol..

[25] Mazidi M., Gao H.-K., Vatanparast H., Kengne A.P. (2017). Impact of the dietary fatty acid intake on C-reactive protein levels in US adults. Medicine.

[26] Julia C., Touvier M., Meunier N., Papet I., Galan P., Hercberg S., Kesse-Guyot E. (2013). Intakes of PUFAs Were Inversely Associated with Plasma C-Reactive Protein 12 Years Later in a Middle-Aged Population with Vitamin E Intake as an Effect Modifier. J. Nutr..

[27] Rangel-Huerta O.D., Aguilera C.M., Mesa M.D., Gil A. (2012). Omega-3 long-chain polyunsaturated fatty acids supplementation on inflammatory biomakers: A systematic review of randomised clinical trials. Br. J. Nutr..

[28] Kiecolt-Glaser J.K., Belury M.A., Andridge R., Malarkey W.B., Hwang B.S., Glaser R. (2012). Omega-3 supplementation lowers inflammation in healthy middle-aged and older adults: A randomized controlled trial. Brain Behav. Immun..

[29] Ferrucci L., Cherubini A., Bandinelli S., Bartali B., Corsi A., Lauretani F., Martin A., Andres-Lacueva C., Senin U., Guralnik J.M. (2006). Relationship of Plasma Polyunsaturated Fatty Acids to Circulating Inflammatory Markers. J. Clin. Endocrinol. Metab..

[30] Perreault M., Roke K., Badawi A., Nielsen D.E., Abdelmagid S.A., El-Sohemy A., Ma D., Mutch D. (2013). Plasma Levels of 14:0, 16:0, 16:1n-7, and 20:3n-6 are Positively Associated, but 18:0 and 18:2n-6 are Inversely Associated with Markers of Inflammation in Young Healthy Adults. Lipids.

[31] Willett W. (1998). Nutritional Epidemiology.

[32] Hodson L., Skeaff C.M., Fielding B.A. (2008). Fatty acid composition of adipose tissue and blood in humans and its use as a biomarker of dietary intake. Prog. Lipid Res..

[33] Hart M.J., Torres S.J., McNaughton S.A., Milte C.M. (2021). Dietary patterns and associations with biomarkers of inflammation in adults: A systematic review of observational studies. Nutr. J..

[34] Barbaresko J., Koch M., Schulze M.B., Nöthlings U. (2013). Dietary pattern analysis and biomarkers of low-grade inflammation: A systematic literature review. Nutr. Rev..

[35] Parkinson L., Cicerale S. (2016). The Health Benefiting Mechanisms of Virgin Olive Oil Phenolic Compounds. Molecules.

[36] Pedret A., Catalán U., Fernández-Castillejo S., Farràs M., Valls R.-M., Rubió L., Canela N., Aragonés G., Romeu M., Castañer O. (2015). Impact of Virgin Olive Oil and Phenol-Enriched Virgin Olive Oils on the HDL Proteome in Hypercholesterolemic Subjects: A Double Blind, Randomized, Controlled, Cross-Over Clinical Trial (VOHF Study). PLoS ONE.

[37] Murff H.J., Edwards T.L. (2014). Endogenous Production of Long-Chain Polyunsaturated Fatty Acids and Metabolic Disease Risk. Curr. Cardiovasc. Risk Rep..

