# Supplementary material for: Red Blood Cell Membrane Fatty Acid Composition, Dietary Fatty Acid Intake and Diet Quality as Predictors of Inflammation in a Group of Australian Adults
Source: Nutrients. 2023 May 21;15(10):2405. doi: 10.3390/nu15102405 (PMC10223124; doi:10.3390/nu15102405)
Supplement: Supplementary file 1 [file nutrients-15-02405-s001.zip › nutrients-2389323-supplementary.pdf]

**Supplementary Table S1.** Adjusted and unadjusted mixed-effects models showing the relationship between red blood cell and dietary fatty acids and inflammatory markers.

| | Unadjusted | | Simple Adjustment [(a)] | | Multiple Adjustments [(b)] | |
|---|---|---|---|---|---|---|
| | **β (95% CI)** | **p-value** | **β (95% CI)** | **p-value** | **β (95% CI)** | **p-value** |
| **IL-6 & Fatty Acids** | | | | | | |
| RBC - SFA | 0.25 (-0.09, 0.60) | 0.15 | 0.19 (-0.16, 0.54) | 0.29 | -0.08 (-0.47, 0.31) | 0.68 |
| RBC – MUFA | -0.07 (-0.44, 0.29) | 0.70 | -0.05 (-0.42, 0.31) | 0.78 | 0.36 (-0.05, 0.78) | 0.08 |
| RBC – n-6 PUFA | -0.20 (-0.47, 0.07) | 0.15 | -0.19 (-0.46, 0.08) | 0.17 | -0.08 (-0.36, 0.20) | 0.59 |
| RBC – n-3 PUFA | 0.13 (-0.22, 0.48) | 0.46 | 0.17 (-0.18, 0.52) | 0.33 | -0.05 (-0.41, 0.30) | 0.77 |
| RBC – n-3 Index | 0.06 (-0.34, 0.45) | 0.76 | 0.11 (-0.28, 0.50) | 0.59 | -0.13 (-0.51, 0.25) | 0.50 |
| Dietary Saturated Fat intake | 0.03 (-0.01, 0.07) | 0.11 | 0.03 (-0.01, 0.07) | 0.15 | 0.03 (-0.02, 0.07) | 0.22 |
| Dietary monounsaturated fat intake | 0.00 (-0.04, 0.05) | 0.88 | 0.00 (-0.05, 0.04) | 0.89 | 0.01 (-0.03, 0.06) | 0.59 |
| Dietary polyunsaturated fat intake | -0.03 (--0.17, 0.11) | 0.67 | -0.05 (-0.19, 0.08) | 0.46 | 0.01 (-0.14, 0.16) | 0.91 |
| Dietary n-3 intake | 0.00 (-0.003, 0.002) | 0.55 | 0.00 (-0.003, 0.002) | 0.62 | 0.00 (-0.003, 0.002) | 0.66 |
| **TNF-$\alpha$ & RBC Fatty Acids** | | | | | | |
| RBC - SFA | 0.02 (-0.02, 0.05) | 0.44 | 0.00 (-0.04, 0.04) | 0.93 | 0.00 (-0.05, 0.04) | 0.85 |
| RBC – MUFA | 0.01 (-0.05, 0.06) | 0.81 | 0.02 (-0.04, 0.07) | 0.54 | 0.03 (-0.04, 0.09) | 0.39 |
| RBC – n-6 PUFA | -0.01 (-0.05, 0.03) | 0.51 | -0.01 (-0.05, 0.03) | 0.71 | 0.00 (-0.04, 0.04) | 0.95 |
| RBC – n-3 PUFA | -0.02 (-0.07, 0.04) | 0.61 | -0.01 (-0.07, 0.05) | 0.79 | -0.02 (--0.09, 0.04) | 0.50 |
| RBC – n-3 Index | -0.02 (-0.09, 0.04) | 0.46 | -0.01 (-0.09, 0.05) | 0.66 | -0.03 (-0.10, 0.04) | 0.44 |
| Dietary Saturated Fat intake | **0.01 (0.001, 0.01)** | **0.02** | **0.01 (0.001, 0.01)** | **0.03** | **0.001 (0.001, 0.01)** | **0.02** |
| Dietary monounsaturated fat intake | 0.00 (-0.002, 0.01) | 0.21 | 0.00 (-0.003, 0.01) | 0.30 | 0.00 (-0.003, 0.01) | 0.28 |
| Dietary polyunsaturated fat intake | 0.00 (-0.01, 0.02) | 0.63 | 0.00 (-0.01, 0.02) | 0.78 | 0.01 (-0.01, 0.02) | 0.55 |
| Dietary n3 intake | 0.00 (-0.0004, 0.0002) | 0.46 | 0.00 (-0.0004, 0.0002) | 0.48 | 0.00 (-0.001, 0.0002) | 0.20 |
| **CRP & RBC Fatty Acids** | | | | | | |
| RBC – SFA | **0.55 (0.06, 1.05)** | **0.03** | **0.51 (0.01, 1.02)** | **0.047** | 0.30 (-0.27, 0.88) | 0.30 |
| RBC – MUFA | **-0.88 (-1.37, -0.39)** | **<0.001*** | **-0.87 (-1.37, -0.38)** | **<0.001*** | -0.59 (-1.26, 0.09) | 0.09 |
| RBC – n-6 PUFA | -0.07 (-0.48, 0.34) | 0.73 | -0.06 (-0.47, 0.35) | 0.77 | -0.07 (-0.54, 0.41) | 0.79 |
| RBC – n-3 PUFA | 0.34 (-0.18, 0.86) | 0.20 | 0.38 (-0.15, 0.90) | 0.16 | 0.21 (-0.41, 0.83) | 0.51 |
| RBC – n-3 Index | 0.46 (-0.12, 1.04) | 0.12 | 0.51 (-0.07, 1.09) | 0.08 | 0.35 (-0.32, 1.02) | 0.31 |
| Dietary Saturated Fat intake | -0.02 (-0.08, 0.04) | 0.52 | -0.02 (-0.08, 0.04) | 0.48 | -0.02 (-0.09, 0.05) | 0.64 |
| Dietary monounsaturated fat intake | -0.04 (-0.11, 0.02) | 0.18 | -0.05 (-0.11, 0.01) | 0.13 | -0.02 (-0.10, 0.05) | 0.52 |

| | | | | | | |
|---|---|---|---|---|---|---|
| Dietary polyunsaturated fat intake | **-0.21 (-0.40, -0.01)** | **0.04** | **-0.23 (-0.42, -0.03)** | **0.02** | -0.12 (-0.35, 0.12) | 0.33 |
| Dietry n-3 Intake | -0.003 (-0.01, 0.001) | 0.09 | -0.003 (-0.01, 0.001) | 0.10 | -0.003 (-0.01, 0.001) | 0.15 |

Data is presented for mixed effects models with beta-coefficients, 95% CI and p-values. Findings with $p$ <0.05 are considered statistically significant and bolded.

(a) adjusted for time only

(b) adjusted for age, BMI, sex, time, intake of anti-inflammatory supplements, inflammatory conditions and smoking status

* Remained significant when adjusting for multiple testing using Bonferroni correction.

CRP, C-reactive protein; IL-6, interleukin 6; MUFA, monounsaturated fatty acids; n-3, omega-3; PUFA, polyunsaturated fatty acids; RBC, red blood cell; SFA, saturated fatty acids; TNF-$\alpha$, tumour necrosis factor $\alpha$.

**Supplementary Table S2:** Adjusted and unadjusted mixed-effects models showing the relationship between individual red blood cell membrane fatty acids and inflammatory markers.

| | Unadjusted | | Adjusted [(a)] | | Adjusted [(b)] | |
|---|---|---|---|---|---|---|
| | **β (95% CI)** | **p-value** | **β (95% CI)** | **p-value** | **β (95% CI)** | **p-value** |
| **IL-6 & Fatty Acids** | | | | | | |
| C14:0 | .18 | 0.81 | .04 | 0.95 | -.75 | 0.33 |
| C16:0 | .32 | 0.24 | .19 | 0.50 | -.10 | 0.71 |
| C18:0 | .42 | 0.14 | .34 | 0.24 | -.17 | 0.59 |
| C18:1n-9 | -.17 | 0.48 | -.19 | 0.42 | -.02 | 0.93 |
| C18:1n-7 | .68 | 0.39 | .83 | 0.29 | **1.61** | **0.04** |
| C18:2n-6 | -.20 | 0.35 | -.20 | 0.34 | .14 | 0.50 |
| C20:0 | -1.53 | 0.30 | -1.47 | 0.32 | -1.22 | 0.39 |
| C20:1n-9 | -1.46 | 0.58 | -1.91 | 0.46 | -1.23 | 0.63 |
| C20:2n-6 | -1.65 | 0.49 | -1.62 | 0.49 | -.85 | 0.71 |
| C20:3n-6 | .35 | 0.27 | .43 | 0.16 | **.63** | **0.04** |
| C20:4n-6 | -.13 | 0.44 | -.15 | 0.38 | -.25 | 0.15 |
| C20:5n-3 | .27 | 0.59 | .50 | 0.32 | .05 | 0.91 |
| C22:0 | -.16 | 0.83 | .10 | 0.89 | .66 | 0.39 |
| C22:4n-6 | **-.86** | **0.048** | **-.90** | **0.04** | **-.89** | **0.04** |

| | | | | | | |
|---|---|---|---|---|---|---|
| C22:5n-3 | .75 | 0.17 | .77 | 0.15 | .45 | 0.42 |
| C22:6n-3 | .03 | 0.89 | .07 | 0.78 | -.14 | 0.56 |
| C24:0 | -.09 | 0.80 | 0.00 | 0.99 | .25 | 0.48 |
| C24:1n-9 | -.01 | 0.99 | 0.06 | 0.85 | .42 | 0.18 |
| **TNF-α & RBC Fatty Acids** | | | | | | |
| C14:0 | -.01 | 0.88 | -.05 | 0.55 | -.06 | 0.46 |
| C16:0 | .01 | 0.75 | -.02 | 0.53 | -.02 | 0.57 |
| C18:0 | .05 | 0.12 | .03 | 0.38 | -.01 | 0.87 |
| C18:1n-9 | .02 | 0.56 | .02 | 0.57 | .00 | 0.95 |
| C18:1n-7 (Vaccenate) | .00 | 0.96 | .03 | 0.76 | .10 | 0.24 |
| C18:2n-6 | -.03 | 0.38 | -.03 | 0.45 | -.02 | 0.52 |
| C20:0 | -.07 | 0.64 | -.05 | 0.74 | -.05 | 0.76 |
| C20:1n-9 | .25 | 0.32 | .19 | 0.44 | .19 | 0.43 |
| C20:2n-6 | .20 | 0.38 | .21 | 0.34 | .22 | 0.30 |
| C20:3n-6 | -.01 | 0.89 | .00 | 0.97 | .05 | 0.21 |
| C20:4n-6 | .00 | 0.86 | .01 | 0.77 | .00 | 0.85 |
| C20:5n-3 | -.12 | 0.08 | -.08 | 0.23 | -.10 | 0.17 |
| C22:0 | -.10 | 0.18 | -.08 | 0.33 | -.04 | 0.61 |
| C22:4n-6 | -.11 | 0.12 | -.11 | 0.11 | -.10 | 0.18 |
| C22:5n-3 | .03 | 0.69 | .02 | 0.72 | .01 | 0.92 |
| C22:6n-3 | .01 | 0.88 | .01 | 0.77 | .00 | 0.94 |
| C24:0 | .01 | 0.79 | .03 | 0.50 | .05 | 0.24 |
| C24:1n-9 | -.02 | 0.67 | .00 | 0.95 | .03 | 0.47 |
| **CRP & RBC Fatty Acids** | | | | | | |
| C14:0 | .48 | 0.66 | .41 | 0.71 | -.07 | 0.95 |
| C16:0 | **1.27** | **0.001** | **1.19** | **0.002** | **.98** | **0.02** |
| C18:0 | .60 | 0.14 | .55 | 0.19 | -.07 | 0.89 |
| C18:1n-9 | -.42 | 0.23 | -.44 | 0.22 | -.11 | 0.79 |
| C18:1n-7 | **-3.65** | **0.001** | **-3.53** | **0.001** | **-2.56** | **0.02** |

| | | | | | | |
|---|---|---|---|---|---|---|
| C18:2n-6 | **-.80** | **0.01** | **-.79** | **0.01** | -.65 | 0.06 |
| C20:0 | -1.05 | 0.62 | -1.14 | 0.59 | -1.22 | 0.56 |
| C20:1n-9 | -2.25 | 0.55 | -2.58 | 0.49 | -1.73 | 0.63 |
| C20:2n-6 | -2.74 | 0.43 | -2.63 | 0.44 | -1.46 | 0.65 |
| C20:3n-6 | **-.94** | **0.04** | -.85 | 0.06 | -.64 | 0.20 |
| C20:4n-6 | **.61** | **0.01** | **.59** | **0.02** | .41 | 0.13 |
| C20:5n-3 | .79 | 0.29 | 1.06 | 0.16 | 1.11 | 0.18 |
| C22:0 | -1.36 | 0.20 | -1.04 | 0.34 | -.49 | 0.67 |
| C22:4n-6 | .52 | 0.45 | .47 | 0.49 | .55 | 0.46 |
| C22:5n-3 | -.31 | 0.70 | -.34 | 0.68 | -.77 | 0.39 |
| C22:6n-3 | .57 | 0.13 | .61 | 0.10 | .36 | 0.37 |
| C24:0 | -.39 | 0.42 | -.35 | 0.48 | -.08 | 0.88 |
| C24:1n-9 | **-1.03** | **0.02** | **-1.01** | **0.02** | -.55 | 0.26 |

Data is presented for mixed effects models with beta-coefficients, 95% CI and p-values. Findings with $p < 0.05$ are considered statistically significant and bolded.

(a) adjusted for time only

(b) adjusted for age, BMI, sex, time, intake of anti-inflammatory supplements, inflammatory conditions and smoking status

CRP, C-reactive protein; IL-6, interleukin 6; RBC, red blood cell; TNF-$\alpha$, tumour necrosis factor $\alpha$.
